# Constructing Invisible Walls through National and Global Policy

**DOI:** 10.3390/children6070083

**Published:** 2019-07-17

**Authors:** Stephanie Ettinger de Cuba, Allison Bovell-Ammon, Diana Becker Cutts

**Affiliations:** 1 Children’s HealthWatch—Department of Pediatrics, Boston University School of Medicine, One Boston Medical Center Place, Vose Hall 5th floor, Boston, MA 02118, USA; 2 Children’s HealthWatch—Department of Pediatrics, Boston Medical Center, One Boston Medical Center Place, Vose Hall 4th floor, Boston, MA 02118, USA; 3 Hennepin County Medical Center, Department of Pediatrics, G7, 701 Park Avenue, Minneapolis, MN 55415, USA

**Keywords:** climate change, migration, immigration policy, children’s rights

## Abstract

Worldwide 37,000 people are forced to flee their homes every day due to conflict and persecution. The factors that lead people to leave their home countries often originate with economic deprivation and violence, escalated to a level that becomes a struggle for survival. Climate change, as it has accelerated over the last three to four decades and negatively impacted natural resources, contributes to a parallel increase in strife and migration. The US response to migration has been to construct an “Invisible Wall” of isolationist and xenophobic policies, many of which are especially harmful to children and their families. The southern US border is perhaps the most high profile location of the Invisible Wall’s construction, fortified by federal policies and a withdrawal from international cooperation. Global reengagement on climate change and migration, US ratification of the Convention on the Rights of the Child, and destruction of the Invisible Wall will help to create a world where children can thrive.

Worldwide 37,000 people are forced to flee their homes every day due to conflict and persecution, according to the United Nations (UN) High Commissioner for Refugees [1]. The global levels of forcibly displaced people are the highest ever on record, numbering nearly 71 million people in turmoil from all corners of the globe, including Syria, Afghanistan, and South Sudan as well as Southeast Asia and Central and South America [1].

The factors that lead people to flee their home countries often originate with economic deprivation and violence, escalated to a level that becomes a struggle for survival. Whether the conflict is between ethnic or religious groups, law abiding citizens and gangs, or rival governments, many are rooted in a lack of equitable distribution of resources and an inability for people to generate income and wealth [2,3]. As competition for resources and power fans tension, scapegoats are sought, often fueling the blame of already historically oppressed groups [4]. Climate change, as it has accelerated over the last three to four decades and negatively impacted natural resources, contributes to a parallel increase in strife; Syria’s civil war was sparked in part by a severe four-year drought causing 1.5 million people to migrate from rural areas to cities, increasing poverty and unrest^4^. In Central America, historical tensions over land ownership and rights were partially responsible for severe economic deprivation for large parts of the population and further exacerbated by US corporate and political involvement. Here, too, climate change plays a role. The “Central American Dry Corridor” is a zone that has been experiencing severe drought and floods, making it nearly impossible for families to earn a livelihood [5]. This extreme hardship fuels today’s widespread violence and subsequent mass migration. In fact, the link between climate change and violence is now a demonstrated fact [6,7]. A systematic review of the global evidence found that collective violence and climate change, particularly when experienced as higher temperatures and extreme levels of precipitation, were repeatedly causally associated [7].

When climate change, economic deprivation, and violence come together, staying put becomes untenable, especially for parents and guardians desperate to provide for their children. Our research at Children’s HealthWatch demonstrates over and over again that when parents lack the resources to provide basic needs for their children, they will go to great lengths of self-deprivation and personal sacrifice in order to protect their children from hardship [8]. The chance of a better future elsewhere, when there is none available otherwise, leads to a forced choice to risk all and flee. Each person’s story of displacement is unique but all share fundamental characteristics—fear for their lives and livelihoods and the search for peace and stability [9]. With millions of people on the move, it is all too easy to get lost in the staggering numbers. The world was reminded again of the very human toll recently with the widely publicized photo of Óscar Alberto Martínez Ramírez and his toddler daughter, Valeria, face down in the Rio Grande after being swept away by the current when—his family unable to obtain an appointment for asylum with United States (US) authorities—they attempted to cross the river to the US [10]. This painful image reminded the world of the agonizingly similar photo of Alan Kurdi, half a globe away, whose drowned body washed up on the beaches of Turkey after his family attempted to sail to Greece for asylum.

What of such children? Are these sad images isolated cases or do they speak to more fundamental issues? According to the UN Office of the High Commissioner on Human Rights’ Convention on the Rights of the Child, children are owed a special responsibility “by reason of (their) physical and mental immaturity, need special safeguards and care, including appropriate legal protection, before as well as after birth [11].” In addition, these special rights include those who are seeking refugee status—they should, “whether unaccompanied or accompanied by his or her parents or by any other person, receive appropriate protection and humanitarian assistance,” including “protect(ing) and assist(ing) such a child and…trac(ing) the parents or other members of the family of any refugee child in order to obtain information necessary for reunification with his or her family [11].” The United States has not lived up to this international code of responsibility—notably never ratifying the convention. Far from providing protection, humanitarian assistance, special safeguards, or assistance in reuniting families, the US government has made it official government policy to do the opposite. Reports from the southern border offer horrific stories of extreme neglect, unsanitary conditions, emotional and physical cruelty, and unnecessary and cruel family separation—all under orders from US Customs and Border Protection, which falls under the US Department of Homeland Security (DHS) [12,13]. Recent revelations of a secret Facebook group where Border Patrol members’ racist and misogynist posts taunted migrants and officials in opposition to their actions and celebrated deaths and cruelty [14] were dismissed as “a couple of bad apples” by DHS’ Acting Secretary [15]. However, with approximately 9500 members and Border Patrol leadership aware of the group for the past three years, that explanation defies credibility.

Beyond the present horrifying circumstances of the children and families, the harms being perpetrated are long-lasting and deep at both an individual and policy level. Neuroscientists from around the world have documented the fundamental and permanent brain changes that occur in children who have experienced upheaval, violence, and trauma, altering long-term socio-emotional well-being and ability to form attachments [16,17,18,19,20,21]. Their future, adult physical health is also threatened by significantly increased risk of poor cardiovascular health, acceleration of age-related disease onset, and early mortality [19,22]. These assaults on health put an entire generation of children at risk, digging a deeper hole of despair for their families.

Also disturbing is policy violence—a term referencing the effect of legislative, regulatory, and other types of policy decisions on people living in poverty [23]. The policy violence in place at the US southern border is part of a systematic effort to build an “Invisible Wall” [24]. The border is perhaps the most high profile location of its construction, but the source of its fortification includes policies that span DHS and the State Department to the Department of Housing and Urban Development, the Social Security Administration, the Department of Justice, and the Department of Agriculture. Federal agencies’ explicit mandate from the White House has been to report on steps the agencies have taken to comply with laws restricting immigrants’ eligibility for benefits, which benefits they administer are means-tested, and whether any other benefits they provide should be considered means-tested. Participation in means-tested programs have been proposed as a way to determine ineligibility for entry into the US and for adjustment of status once present (known as being a “public charge”) [24,25]. These organized efforts, among others, are designed to make it dramatically more difficult for immigrants, refugees, and asylees to enter the country legally, extend a visa, receive assistance and be counted in the Census, apply for a ‘green card’ (Legal Permanent Residency), and apply for citizenship. The fundamental message being broadcast is “you are not welcome here.” Given the zeal with which the changes are being applied to black and brown immigrants, it is an inescapable conclusion that racism and discrimination are powerful motivating forces [26,27].

At its core, these policy efforts are also a return to isolationism, a position that was rejected decades ago by world powers. This new embrace of isolationist, xenophobic stances, which have been supported by leaders in the US as well as other countries around the world [9], regresses us globally to a time that no longer exists, if such a time ever did. In fact, if we seek a world where children are able to thrive, the way forward can only be achieved in cooperation with others. The US once prided itself on its diplomatic place in the world as a partner and negotiator in multilateral efforts that helped bring peace. The professionalism of the diplomatic corps was revered and the US came to be seen as a moral authority. That position has crumbled with the withdrawal from international treaties like the Paris Climate Accord and mercurial stances on foreign and immigration policies.

An alternative approach, reengagement at the global level, is an opportunity to regain our footing and move toward real and lasting solutions of the twin challenges of our time—international migration and climate change. As leaders of small countries in the Pacific Islands have been teaching the world, the effect of climate change disproportionately places the greatest burden and cost on our world’s poorest people [28], exacerbating global inequality [29]. Without collective effort, worsening conditions in poor countries will only spur further migration as heat increases crop failure, economic hardship, and violence. This fall, the UN will convene a global climate summit in New York. Other countries from Japan, to Germany, to the United Kingdom are making bold commitments to change [30]. If the US joined the fight, how much more progress could we make and how much more suffering could we avert—at home and abroad? Current US government policy calls on our darkest demons, but it does not have to be so—policy can be a force for dramatic and lasting good, creating a world where children can thrive. The US should reengage with the global community, finally ratify the Convention on the Rights of the Child, shoulder its share of responsibility to slow climate change and thus also migration pressures, immediately institute humanitarian standards for treatment of migrants, particularly children, and tear down the “Invisible Wall” [24].

## References

[1] (2019). Figures at a Glance—Statistical Yearbooks. https://www.unhcr.org/en-us/figures-at-a-glance.html.

[2] Orellana López A. (2015). Bolivia, 15 Years on from the Water War. https://democracyctr.org/article/bolivia-15-years-on-from-the-water-war/.

[3] Al-Jazeera Syria’s Civil War Explained from the Beginning: On March 15, the War Entered Its Eighth Year, 14 April 2019. https://www.aljazeera.com/news/2016/05/syria-civil-war-explained-160505084119966.html.

[4] Batware B. (2012). Rwandan Ethnic Conflict: A Historical Look at Root Causes.

[5] The Climate Reality Project (2019). How the Climate Crisis Is Driving Central American Migration. https://www.climaterealityproject.org/blog/how-climate-crisis-driving-central-american-migration.

[6] Miles-Novelo A., Anderson C.A. (2019). Climate change and psychology: Effects of rapid global warming on violence and aggression. Curr. Clim. Chang. Reports.

[7] Levy B.S., Sidel V.W., Patz J.A. (2017). Climate change and collective violence. Annu. Rev. Public Health.

[8] Frank D.A., Casey P.H., Black M.M., Rose-Jacobs R., Chilton M., Cutts D., March E., Heeren T., Coleman S., Ettinger de Cuba S. (2010). Cumulative hardship and wellness of low-income, young children: Multisite surveillance study. Pediatrics.

[9] Sweetland Edwards H. (2019). The Stories of Migrants Risking Everything for a Better Life. Time Magazine.

[10] Aguilera J. (2019). How Controversy Over the Photo of a Drowned Migrant Father and Daughter Captured the Profound Cost of the Border Crisis. Time Magazine.

[11] (1989). Convention on the Rights of the Child. https://www.ohchr.org/en/professionalinterest/pages/crc.aspx.

[12] Fetters A. (2018). The exceptional cruelty of a no-hugging policy. Atlantic.

[13] Raff J. (2019). What a Pediatrician Saw Inside a Border Patrol Warehouse. Atlantic.

[14] Thompson A.C. (2019). Inside the Secret Border Patrol Facebook Group Where Agents Joke About Migrant Deaths and Post Sexist Memes. ProPublica.

[15] (2019). Acting Secretary McAleenan Appears on Fox News Channel to Discuss Crisis on the Border. https://www.dhs.gov/news/2019/07/05/acting-secretary-mcaleenan-appears-fox-news-channel-discuss-crisis-border.

[16] Devakumar D., Birch M., Osrin D., Sondorp E., Wells J.C.K. (2014). The intergenerational effects of war on the health of children. BMC Medicine.

[17] Wright M.O., Master A.S., Hubbard J.J., Cicchetti D., Toth S.L. (1997). Long-Term Effects of Massive Trauma: Developmental and Psychobiological Perspectives. Developmental Perspectives on Trauma: Theory, Research, and Intervention.

[18] Macksoud M.S., Dyregrov A., Raundalen M., Wilson J.P., Raphael B. (1993). Traumatic War Experiences and Their Effects on Children. International Handbook of Traumatic Stress Syndromes.

[19] Locke C., Southwick K., McCloskey L., Fernández-Esquer M. (1996). The psychological and medical sequelae of war in Central American refugee mothers and children. Arch. Pediatr. Adolesc. Med..

[20] Hoksbergen R., ter Laak J., Van Dijkum C., Rijk S., Rijk K., Stoutjesdijk F. (2003). Posttraumatic stress disorder in adopted children from Romania. Am. J. Orthopsychiatry.

[21] Goldstein R., Wampler N., Wise P.H. (1997). War experiences and distress symptoms of Bosnian children. Pediatrics.

[22] Ahmadi N., Hajsadeghi F., Mirshkarlo H.B., Budoff M., Yehuda R., Ebrahimi R. (2011). Post-traumatic stress disorder, coronary atherosclerosis, and mortality. Am. J. Cardiol..

[23] Kaufmann G. (2018). The Poor People’s Campaign Calls Out ‘Policy Violence’. The Nation.

[24] (2019). Protecting Immigrant Families Campaign. https://protectingimmigrantfamilies.org/analysis-research/.

[25] Rose J. (2019). Family Separations Under “Remain in Mexico” Policy. https://www.npr.org/2019/07/05/738860155/family-separations-under-remain-in-mexico-policy.

[26] Farias C. (2019). Is There Racist Intent Behind the Census Citizenship Question?. New Yorker.

[27] (2019). Understanding Trump’s Muslim Ban. https://www.nilc.org/issues/immigration-enforcement/understanding-the-muslim-bans/.

[28] Worland J. (2019). The Leaders of These Sinking Countries Are Fighting to Stop Climate Change. Here’s What the Rest of the World Can Learn. Time Magazine.

[29] Worland J. (2019). Climate Change Has Already Increased Global Inequality. It Will Only Get Worse. Time Magazine.

[30] Worland J. (2019). U.N. Head: Climate Change Can Prove the Value of Collective Action. Time Magazine.

